# Role of Buffer Layers in Defect Chemistry and Parasitic Phase Formation of BiFeO_3_ Films on Silicon

**DOI:** 10.1021/acsomega.5c08852

**Published:** 2026-01-29

**Authors:** Saleh H. Fawaeer, Wala’ M. Al-Qaisi, Vlasta Sedláková, Marwan S. Mousa, Alexandr Knápek, Dinara Sobola

**Affiliations:** † Central European Institute of Technology, 613011 Brno University of Technology, Purkyňova 123, Brno 612 00, Czech Republic; ‡ Department of Physics, Faculty of Electrical Engineering and Communications, Brno University of Technology, Technická 2848/8, Brno 616 00, Czech Republic; § Department of Renewable Energy Engineering, 144851 Jadara University, Irbid 21110, Jordan; ∥ 652457 Institute of Scientific Instruments of Czech Academy of Sciences, Královopolská 147, Brno 612 00, Czech Republic; ⊥ Institute of Physics of Materials, Czech Academy of Sciences, Žižkova 22, Brno 616 62, Czech Republic

## Abstract

Achieving the reliable integration of bismuth ferrite with silicon requires precise control over phase formation, cation stoichiometry, and near-surface oxygen chemistry. In this study, BiFeO_3_ films were deposited by pulsed laser deposition onto Ti- and TiO_2_-buffered Si substrates under varied oxygen partial pressures and substrate temperatures. Structural, morphological, and chemical evolutions were investigated using X-ray diffraction, scanning electron microscopy, and combined survey and high-resolution X-ray photoelectron spectroscopy. Both buffer types yield polycrystalline BiFeO_3_ films; Ti-buffered samples exhibit lower variations in Bi/Fe surface ratios, whereas TiO_2_buffered films show a reduced contribution from hydroxyl-related oxygen species at the surface. X-ray photoelectron spectroscopy confirms that Bi and Fe remain exclusively in the trivalent state under all growth conditions. High-resolution oxygen spectra demonstrate that oxygen chemistry is the most sensitive indicator of near-surface disorder, reflecting contributions from lattice oxygen and surface hydroxylation arising from ambient exposure. Minor Bi_2_O_3_ phases persist across the investigated deposition window; however, their evolution, together with surface oxygen trends, indicates that intermediate-to-high substrate temperatures combined with moderate-to-low oxygen pressures provide the most favorable conditions for stabilizing near-stoichiometric BiFeO_3_. Overall, the results highlight oxide buffer layers as effective regulators of surface chemistry, enabling a scalable route for integrating BiFeO_3_ films on silicon.

## Graphical abstract

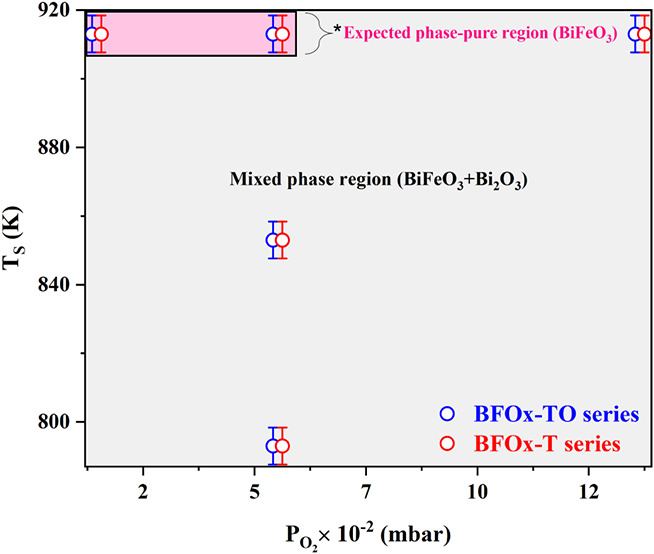

## 1 Introduction

Bismuth ferrite (BiFeO_3_, BFO) is a room-temperature multiferroic that combines robust ferroelectric polarization with antiferromagnetic ordering, making it a promising candidate for multifunctional devices ranging from nonvolatile memory and spintronic logic to energy harvesting and optoelectronics. Its high Curie temperature, substantial polarization, and relatively wide bandgap support operation in harsh environments and motivate its integration with silicon-compatible platforms where multifunctionality and scalable processing are critical.^1–3^

Achieving phase-pure BFO thin films, however, remains challenging due to the narrow thermodynamic stability window of the perovskite phase. The volatility of Bi at elevated substrate temperatures (*T*_S_) and the sensitivity of Bi–O and Fe–O coordination to oxygen partial pressure (*P*_O_2__) frequently result in the formation of impurity and/or secondary bismuth oxide phases, most commonly bismuth oxide (Bi_2_O_3_).^4–8^ Such parasitic phases can complicate film reproducibility, influence leakage pathways, and modify surface chemistry, thereby motivating a detailed investigation into the processing conditions that govern their emergence.

Buffer layers play a key role in determining the BFO phase evolution by influencing interfacial chemistry, surface termination, and strain accommodation. Oxide buffers such as SrRuO_3_, SrTiO_3_, and TiO_2_ have been reported to promote improved crystallinity and stable Bi/Fe valence states, whereas metallic Ti buffers may partially oxidize during high-temperature growth, forming an interfacial TiO_*x*_ layer that modifies nucleation behavior.^9–13^ Despite these advances, direct comparisons between Ti- and TiO_2_-buffered Si substrates under identical *P*_O_2__–*T*_S_ conditions remain limited, especially concerning their ability to regulate the formation and persistence of Bi_2_O_3_.

Most previous studies have focused primarily on optimizing conditions to eliminate impurity phases, while fewer have systematically mapped the deposition conditions under which Bi_2_O_3_ remains stable. Treating parasitic phase formation as an informative diagnostic, rather than a defect to be ignored, provides an alternative route for identifying the thermodynamic and kinetic boundaries that govern BFO stability.^14–18^ Such an “impurity-window” approach can reveal the process space that must be avoided as well as the conditions that converge toward the nominal BFO stoichiometry.

In this work, BiFeO_3_ films were deposited by pulsed laser deposition (PLD) onto Ti/Si and TiO_2_/Si substrates under controlled *P*_O_2__–*T*_S_ conditions to assess the buffer-dependent evolution of the structure, morphology, and surface chemistry. Two series of samples were prepared: one grown at *T*_S_ = 913 K and *P*_O_2__ = 1.3 × 10^–1^ mbar, and the other grown at *P*_O_2__ = 5.2 × 10^–2^ mbar with *T*_S_ = 853 and 793 K. X-ray diffraction (XRD), field-emission scanning electron microscopy (FE-SEM), and X-ray photoelectron spectroscopy (XPS) were used to cross-evaluate the phase formation, surface stoichiometry, and oxygen coordination environments. Rather than focusing solely on producing impurity-free films, the evolution of Bi_2_O_3_ is used as a stabilizing indicator to delineate a practical deposition window. The combined results show that intermediate-to-high substrate temperatures paired with moderate-to-low oxygen pressures provide the most promising regime for approaching phase-pure BiFeO_3_ on silicon. This defect-mapping framework offers insight into buffer-layer effects and provides practical guidelines for improving the reliability of the complex oxide integration with Si.

## 2 Experimental Methods

### 2.1 BFO Film Deposition

(100)-oriented crystalline Si substrates (5 × 5 × 0.5 mm) were cleaned via a 10-min piranha etch (3:1 H_2_SO_4_:H_2_O_2_) followed by a 2-min dip in 2% diluted hydrofluoric acid (HF) to remove native oxide. The substrates were rinsed in deionized water and nitrogen-dried to prevent reoxidation.

Two types of buffer layers were deposited to investigate the influence of the underlying interface on the BiFeO_3_ phase formation. A ∼100 nm Ti layer was employed for the Ti/Si series (BFOx-T) and an ∼65 nm TiO_2_ layer for the TiO_2_/Si series (BFOx-TO). The selected thicknesses ensured complete surface coverage and robust adhesion. These values fall within the established ranges of ∼100–120 nm for Ti and ∼50–70 nm for TiO_2_ buffer layers, which are widely reported in oxide/Si integration studies where compact films provide continuous interfaces, minimized pinhole density, and optimized lattice accommodation.^19–21^ Such thickness is consistent with the prevalent Ti/Si and TiO_2_/Si architectures employed for perovskite and multiferroic oxide films on silicon.^22,23^

Both buffer layers were deposited by magnetron sputtering (BESTEC GmbH) using the Ø2″, 0.125″ thick, 99.99% pure Ti and TiO_2_ targets (Kurt Lesker GmbH). The sputtering chamber was first evacuated to 4.3 × 10^–8^ mbar, followed by a 10-min argon (Ar) presputtering step with the substrate shutter closed to clean the target surface. Ti deposition was performed at room temperature under 15 sccm Ar flow and 1 × 10^–3^ mbar background pressure, using 100 W direct-current (DC) power at 370 V and 270 mA with 10 rpm substrate rotation.^24^ This process yielded a deposition rate of ∼0.30 Å s^–1^ (monitored by quartz crystal microbalance, QCM) and a final thickness of 100.23 ± 3.33 nm after ∼56 min of growth, verified by spectroscopic ellipsometry (SE).^25^ TiO_2_ deposition was conducted under the same temperature and Ar flow conditions but at a slightly higher working pressure of 2 × 10^–3^ mbar, using 70 W radio frequency (RF) power at 240 V with identical substrate rotation.^26,27^ The resulting deposition rate was ∼0.05 Å s^–1^, producing a uniform film thickness of 69.14 ± 2.40 nm after ∼3 h and 37 min, also confirmed by SE.

Polycrystalline BFO films were deposited on both buffer types using a high-vacuum PLD system (TSST) equipped with a KrF excimer laser (λ = 248 nm). A Ø2″, 0.25″ thick Bi_1_._15_FeO_3_ ceramic target (15% excess Bi, SurfaceNet GmbH) was employed to compensate for bismuth volatility during ablation. The chamber was evacuated to a base pressure of 4.8 × 10^–8^ mbar, and the target surface was preablated with 1300 laser pulses at 3 Hz in an ambient oxygen to remove surface contaminants and stabilize plume composition. During film growth, the laser fluence was maintained at ∼2.0 J cm^–2^ with a 5 Hz repetition rate, delivering 10,400 pulses to a 0.107 mm^2^ spot area at 45° incidence, with a target–substrate distance of 55 mm. Substrate heating was achieved by an infrared laser and monitored in situ using a calibrated pyrometer.^28,29^

For both buffer configurations, three deposition conditions were adopted: *P*_O_2__ = 1.3 × 10^–1^ mbar, *T*_S_ = 913 K (BFO1-T, BFO1-TO); *P*_O_2__ = 5.2 × 10^–2^ mbar, *T*_S_ = 853 K (BFO2-T, BFO2-TO); and *P*_O_2__ = 5.2 × 10^–2^ mbar, *T*_S_ = 793 K (BFO3-T, BFO3-TO). Each deposition lasted ∼35 min, yielding films with an average thickness of ∼47 ± 5 nm, as determined by SE, corresponding to an average growth rate of 0.226 ± 0.024 Å s^–1^. After deposition, all samples were cooled at ∼303 K min^–1^ under the same *P*_O_2__. The adopted nomenclature reflects both the buffer type (“T” for Ti, “TO” for TiO_2_) and the sample index (1–3) corresponding to the specific *P*_O_2__–*T*_S_ combination.

### 2.2 BFO Film Characterization

The structural, chemical, and morphological characteristics of the BFO films were comprehensively analyzed using complementary techniques to establish correlations between growth conditions and film quality.

Film surface composition and chemical states were examined by XPS (Kratos AXIS Supra) employing monochromatic Al Kα radiation (hν = 1486.6 eV) under a base pressure of ∼2 × 10^–9^ mbar. Charge neutralization was applied throughout to minimize surface charging effects, and no sputter cleaning was carried out to preserve the native chemical environment. Survey spectra were acquired at 80 eV pass energy with a 1.00 eV step size, whereas high-resolution core-level spectra were collected at 20 eV pass energy and 0.10 eV step size over a 300 × 700 μm^2^ analysis area in normal-emission geometry. The inelastic mean free path was estimated using the TPP-2M model from the NIST database, corresponding to an effective probing depth of ∼6–8 nm.^30^ Spectra were processed in CasaXPS, with binding energies referenced to the C 1s peak at 285.0 eV and quantified using modified Wagner relative sensitivity factors (RSFs).^31–34^ Intensities were normalized to 10^1^ counts to enable a direct comparison across all samples.

Film crystallinity and phase purity were evaluated by XRD using a Rigaku SmartLab diffractometer equipped with a Cu Kα source (λ = 1.5418 Å). Measurements were conducted at 40 kV and 30 mA in parallel beam–plate sample alignment (PB–PSA) geometry.^35^ Diffraction patterns were collected in 2θ–ω coupled scan mode from 18° to 60° with a 0.02° step size and a 2° min^–1^ scan speed. Phase identification was performed using HighScore Plus with the ICDD/JCPDS databases,^36^ and subsequent analysis and peak fitting were conducted in OriginPro.

Film surface morphology was analyzed using an FE-SEM (Verios 460L). High-resolution images were captured at a 50 k× magnification with 5 kV accelerating voltage and 50 pA beam current, using a secondary electron detector to reveal fine topographical and textural details.

## 3 Results and Discussion

### 3.1 XRD-Based Phase and Structural Analysis

Figure 1 presents the 2θ–ω diffraction patterns for BFO films deposited on TiO_2_/Si (BFOx-TO) and Ti/Si (BFOx-T) substrates under three *P*_O_2__–*T*_S_ growth conditions. All films exhibit XRD reflections corresponding to rhombohedral BiFeO_3_ (space group R3̅c), including the (012) reflection near ∼22° and the (104)/(110) doublet around ∼31–32°, consistent with the reference card (ICDD 01-071-2494).^37,38^ Minor reflections appearing at ∼21° and 28° are attributed to Bi_2_O_3_ impurity phase (JCPDS 01-074-1375) and are marked with asterisks (*).^39,40^ Buffer-related reflections are also detected. In films grown on TiO_2_/Si, reflections at ∼25°, 48°, and 55° correspond to the (101)_A_, (200)_A_, and (211)_A_ planes of anatase TiO_2_ (JCPDS 01-071-1168) and are labeled as (S). In Ti/Si-based films, additional reflections at ∼27°, 36°, 40°, 44°, and 54° are assigned to the (110)_R_, (101)_R_, (200)_R_, (210)_R_, and (211)_R_ planes of rutile-type (JCPDS 01-089-0552) and are denoted as (S′).^41,42^ No reflections from the temporary silver adhesive (Ag, JCPDS 00-001-1164) were detected in any of the BFO patterns, confirming its complete removal before analysis.

**Figure 1:**
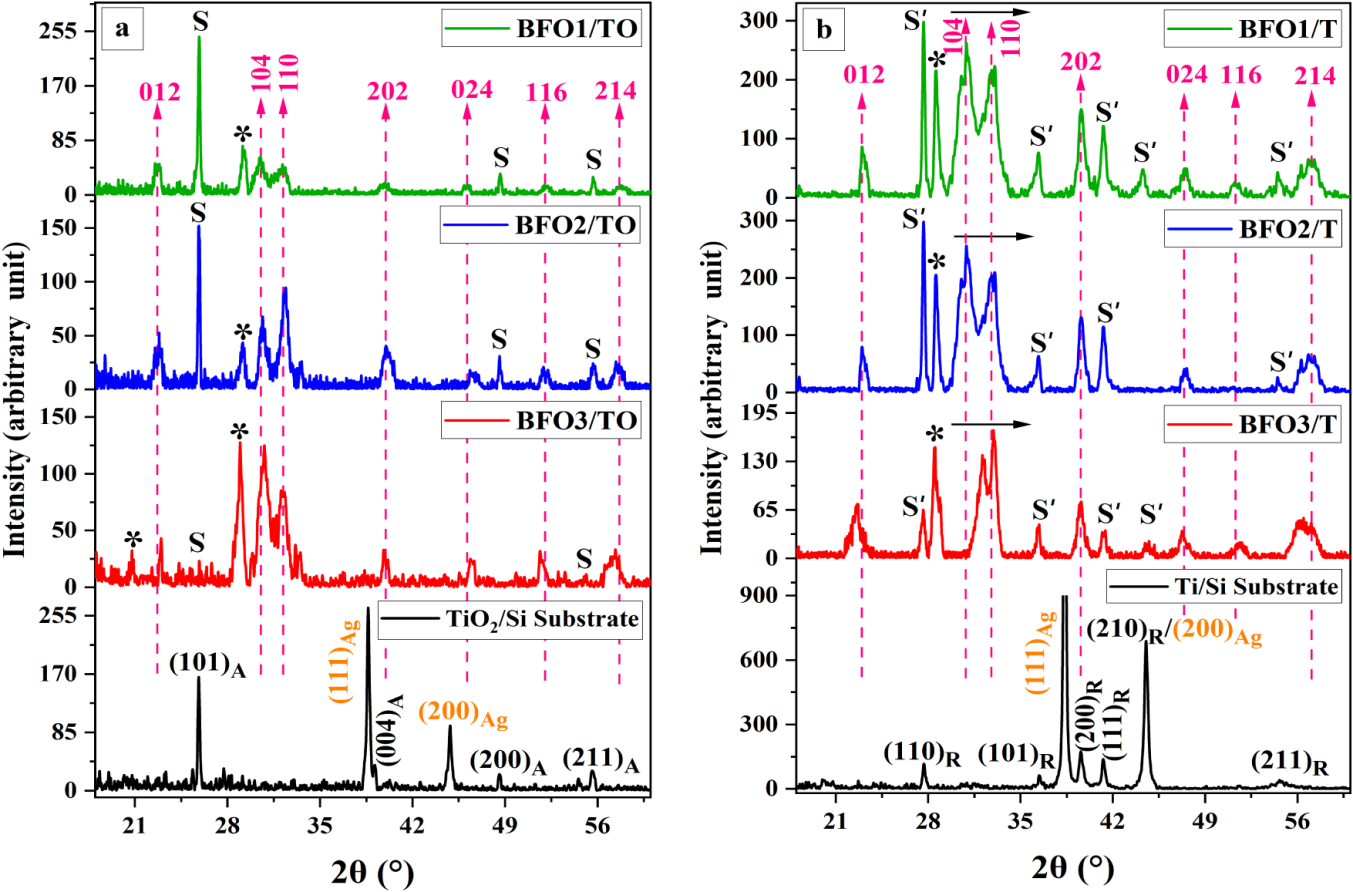
XRD patterns for BFO films grown on (a) TiO_2_/Si (BFOx-TO) and (b) Ti/Si (BFOx-T). Rhombohedral BFO reflections are indexed as Miller indices (hkl) in the hexagonal setting of the R3̅c space group. Reflections corresponding to the Bi_2_O_3_ impurity phase are marked with asterisks (*). Substrate-related reflections are labeled (S) for TiO_2_/Si and (S′) for Ti/Si. Buffer-layer reflections are annotated as (hkl)_A_ for anatase TiO_2_ and (hkl)_R_ for rutile, based on reference scans of PLD-heated, uncleaned substrates. Right-pointing black arrows indicate the shift of the BFO (104)/(110) doublet to higher 2θ in the BFOx-T series relative to that of the BFOx-TO series.

To examine structural trends across deposition conditions, 2θ peak centers and full widths at half-maximum (fwhm) of the BFO (104)/(110) and Bi_2_O_3_ (211) reflections were extracted using pseudo-Voigt fitting line shapes in OriginPro.^43^ Where relevant, instrumental broadening was corrected.^35,44^ These results, together with peak intensities and phase fractions estimated via the single-peak Reference Intensity Ratio (RIR) method, are summarized in Table 1.

**Table 1:** Peak Shape Parameters and Semiquantitative Phase Fractions of BFOx-TO and BFOx-T Samples Derived by the Single-Peak RIR Method^a^

	BiFeO_3_				Bi_2_O_3_		Peak area ratio	RIR method	
Sample	2θ_(104)_ (°)	fwhm (°)	2θ_(110)_ (°)	fwhm (°)	2θ_(211)_ (°)	fwhm (°)	$I_{Bi_{2}O_{3}}/I_{BiFeO_{3}}$	${\left(\%w\mathrm{t.}\right)}_{BiFeO_{3}}$	${\left(\%w\mathrm{t.}\right)}_{Bi_{2}O_{3}}$
**BFO1-TO**	30.70(9)	0.252(7)	32.28(7)	0.248(4)	28.34(9)	0.186(2)	12.6(5)/14.6(2)	74.2 ± 1.1	25.8 ± 1.1
**BFO2-TO**	30.74(6)	0.244(2)	32.30(2)	0.236(9)	28.34(7)	0.178(4)	7.8(3)/22.4(2)	87.7 ± 1.2	12.3 ± 1.2
**BFO3-TO**	30.78(7)	0.247(3)	32.32(3)	0.241(6)	28.31(2)	0.192(3)	24.7(7)/30.8(8)	75.6 ± 1.5	24.4 ± 1.5
**BFO1-T**	30.90(9)	0.293(7)	32.76(5)	0.287(2)	28.33(6)	0.159(1)	34.9(8)/78.2(3)	84.8 ± 0.9	15.2 ± 0.9
**BFO2-T**	30.92(1)	0.285(4)	32.79(2)	0.281(8)	28.33(3)	0.152(5)	31.9(2)/73.2(5)	85.1 ± 1.0	14.9 ± 1.0
**BFO3-T**	32.08(4)	0.238(9)	32.92(7)	0.233(1)	28.30(5)	0.184(4)	27.6(1)/40.3(1)	78.4 ± 1.4	21.6 ± 1.4

a Uncertainties represent ±1σ propagated errors.

For TiO_2_-buffered films (BFOx-TO), a gradual shift of the BFO (104)/(110) reflections toward higher 2θ is observed as the *T*_S_ decreases from 913 to 793 K. This corresponds to a small decrease in interplanar spacing and is consistent with earlier reports on temperature-dependent strain in BFO thin films.^37,45^ In Ti-buffered films (BFOx-T), the BFO reflection positions appear consistently at higher 2θ values compared to the TiO_2_-buffered counterparts under equivalent growth conditions. Similar peak shifts have been reported in BFO grown on metallic or oxygen-reactive bottom electrodes such as Pt or SrRuO_3_.^46–48^ The BFO3-T sample exhibits the largest deviation, where peak positions approach ∼32° and 33°, which may suggest an atypically distorted lattice under this condition, as also observed in the literature under nonequilibrium PLD regimes.^48,49^

The fwhm of the (104) and (110) reflections displays systematic variations across sample sets. Ti-buffered films at higher *T*_S_ (BFO1-T, BFO2-T) show broader peaks (∼0.285–0.293°), whereas TiO_2_-buffered films exhibit narrower peaks (∼0.244–0.252°). This difference is in line with previous studies where peak broadening was associated with increased defect density or reduced coherent scattering domain size.^50^ At 793 K (BFO3-T), the BFO peak width decreases again (∼0.238°), indicating a change in microstructural state relative to higher-temperature growth.^48^

Impurity phase (Bi_2_O_3_) evolution was semiquantified using a single-peak RIR approach based on integrated intensities (peak height × fwhm).^51^ Integrated intensities (integral area) were first normalized by their respective RIR constants and then converted to normalized weight fractions (%wt.) using

1 $$W_{i}=\left(\frac{I_{i}}{{\mathrm{RIR}}_{i}}\times {\left(\sum_{j=1}^{n}\frac{I_{j}}{{\mathrm{RIR}}_{j}}\right)}^{-1}\right)\times \mathrm{100\%}$$

where *W*_*i*_ is the %wt. of the phase *i*, *I*_*i*_ is its integrated intensity, and RIR_***i***_ is the reference intensity. The RIR constants used were 8.70 for BiFeO_3_ and 21.62 for Bi_2_O_3_, taken from the ICDD/JCPDS reference cards.^36,52^

Table 1 shows that Ti-buffered films (BFO1-T, BFO2-T) maintain comparable Bi_2_O_3_ contents (∼15–16 %wt.), while BFO3-T exhibits a moderate increase (∼22 %wt.). In contrast, TiO_2_-buffered films display a stronger dependence on *T*_S_: the lowest Bi_2_O_3_ content (∼12 %wt.) is observed at 853 K (BFO2-TO), whereas both higher (913 K) and lower (793 K) *T*_S_ result in increased Bi_2_O_3_ fractions (∼25–26 %wt.). Similar trends have been reported in the literature, where phase-pure BFO forms only within a narrow *P*_O_2__–*T*_S_ growth window.^7,13,37,53^ Within the present deposition window, three regimes can be distinguished: high *T*_S_/*P*_O_2__—increased Bi volatility and oxidation correlate with higher Bi_2_O_3_ content; intermediate *T*_S_/*P*_O_2__—minimal impurity formation and highest BFO fraction (BFO2-TO/T); low *T*_S_/moderate *P*_O_2__—higher Bi_2_O_3_ content, consistent with incomplete oxide formation and limited structural ordering.^54,55^

These results highlight that the peak position, peak width, and relative phase abundance vary consistently with substrate type and growth conditions. TiO_2_-buffered substrates tend to exhibit sharper BFO reflections and lower impurity levels at intermediate *T*_S_/*P*_O_2__, while Ti-buffered films show broader peaks and relatively stable impurity content across most conditions. In summary, reducing Bi_2_O_3_ formation appears to require avoiding both high-*T*_S_/*P*_O_2__ Bi loss and low-*T*_S_/*P*_O_2__ incomplete phase formation, favoring moderate-to-high *T*_S_ at low-to-moderate *P*_O_2__. These structural observations provide a foundation for subsequent SEM and XPS analyses.

### 3.2 SEM-Based Surface Morphology Analysis

The surface morphology of the BiFeO_3_ films was examined to assess grain shape, surface texture, and microstructural distribution as a function of the buffer layer and deposition conditions. Representative SEM images for TiO_2_-buffered films (BFOx-TO) and Ti-buffered films (BFOx-T) are shown in Figure 2a–c and d–f, respectively.

**Figure 2:**
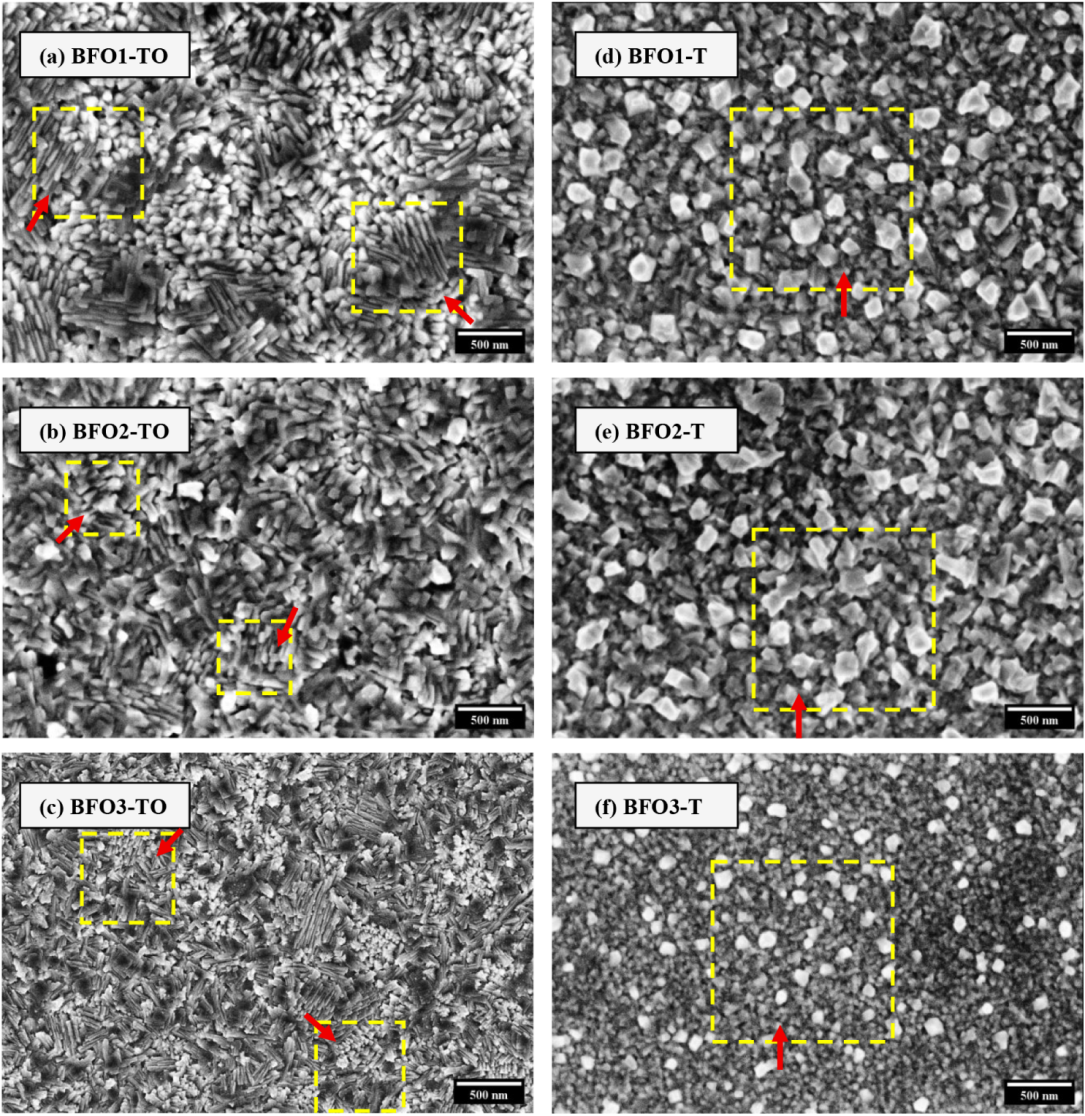
Top-view FE-SEM images of BFO films on TiO_2_/Si (left panels) and Ti/Si (right panels) deposited at (a, d) 913 K, 1.3 × 10^–1^ mbar (BFO1-TO, BFO1-T), (b, e) 853 K, 5.2 × 10^–2^ mbar (BFO2-TO, BFO2-T), and (c, f) 793 K, 5.2 × 10^–2^ mbar (BFO3-TO, BFO3-T). Yellow dashed squares and red arrows in TiO_2_-buffered films highlight elongated grains and locally aligned domains, whereas in Ti-buffered films, they indicate near-equiaxed or polygonal grains. Grain boundary porosities appear as darker contrast features with no visible surface cracks, large voids, or surface Bi_2_O_3_ agglomerates.

All samples grown on TiO_2_/Si exhibit closely packed grains, accompanied by visible porosities at the grain boundaries.^4,56^ In BFO1-TO, the surface is composed of compact grains, some of which are slightly elongated and locally aligned within small domains. Wide dark regions are visible between grains, corresponding to grain boundaries and occasional boundary porosities. In BFO2-TO, the surface becomes more homogeneous, with grain shapes appearing shorter and densely packed compared to those in BFO1-TO. Fewer dark boundary regions are visible. In BFO3-TO, the grains are finer across the entire surface, while dark pits and small void-like separations become slightly more noticeable. No distinct faceted Bi_2_O_3_ clusters or segregated surface phases were observed in any of the TiO_2_-buffered samples.

In contrast, films deposited on Ti/Si predominantly exhibit near-equiaxed or polygonal grains at all *T*_S_.^7,13^ In BFO1-T, the grains are compact, faceted, and well-defined with no large cracks or voids. Dark contrast features at grain boundaries are visible. BFO2-T displays a uniform, fine-grained surface with reduced contrast at grain boundaries and fewer observable gaps between grains. BFO3-T exhibits the smallest grains of the Ti-buffered series, forming a continuous and densely packed surface. The grains are nearly spherical or polygonal with low contrast between adjacent grains. As with TiO_2_-buffered films, no distinct large Bi_2_O_3_ clusters or segregates are visible on the surface.

Across both buffer systems, the average grain size increases with increasing *T*_S_,^57,58^ which is consistent with the XRD results of Table 1. Films grown on TiO_2_/Si display more pronounced grain-shape variations, including elongated features at higher *T*_S_, whereas films on Ti/Si exhibit a predominantly near-equiaxed grain structure at all conditions. In both systems, grain boundaries and occasional boundary porosities are consistently visible as darker contrast features or narrow separations, and no cracks, macroscopic voids, or large impurity-phase agglomerates are present.

### 3.3 XPS-Based Elemental Composition Analysis

Figure 3 presents the normalized XPS survey spectra of the BFO films grown on TiO_2_/Si and Ti/Si substrates under the three *P*_O_2__–*T*_S_ deposition conditions. All spectra display the characteristic Bi 4f, Fe 2p, and O 1s core-level peaks, confirming the presence of the Bi–O and Fe–O bonding environments expected for BiFeO_3_. The measured binding energies (*E*_B_) of Bi 4f (∼158.8 ± 0.4 eV), Fe 2p (∼710.8 ± 0.4 eV), and O 1s (∼529.8 ± 0.4 eV) fall within reported ranges for BFO films.^29,59–61^ A minor C 1s peak at ∼284.8 ± 0.4 eV is also present and attributed to adventitious carbon (AdC).

**Figure 3:**
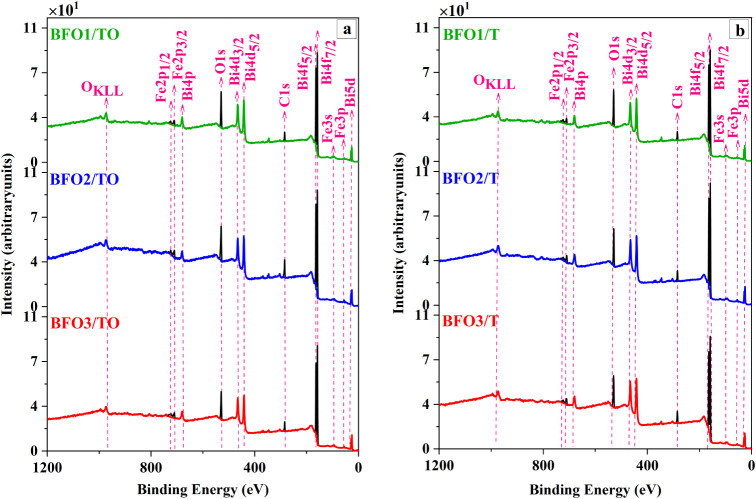
Normalized XPS survey spectra of BFO films deposited on (a) TiO_2_/Si (BFOx-TO) and (b) Ti/Si (BFOx-T). The principal Bi 4f, Fe 2p, O 1s, and C 1s core-level peaks are identified.

Elemental surface compositions were quantified by integrating the survey peak areas after Shirley background subtraction and applying the appropriate RSFs.^32,62^ Since the survey oxygen signal includes contributions from organic oxygen-containing adventitious species, the AdC-correction procedure described in the literature was applied.^63^ In this method, the normalized atomic concentration (%at.) of total survey carbon (C) and the fractional area of C–O functional components in the C 1s spectrum are used to estimate the normalized concentration of O associated with AdC (O_AdC_). Subtracting %at. (O_AdC_) from the total survey oxygen O concentration yields the AdC-corrected oxygen %at. (O_r._), which is more representative of film-related oxygen.^63,64^ The normalized Bi, Fe, O, and C atomic concentrations from survey XPS, together with AdC-corrected O_r._ and the Bi/Fe/O surface elemental ratios for all samples, are summarized in Table 2.

**Table 2:** Survey XPS Surface Compositions in %at. and Bi/Fe/O Surface Elemental Ratios for BFOx-TO and BFOx-T Films (Figure 3)

	Composition (%at.)					Compositional ratios
Sample	Bi	Fe	O	O_r._	C	Bi/Fe/O_r._
**BFO1/TO**	12.4	9.6	50.7	46.5	27.3	1.3:1:4.8
**BFO2/TO**	9.8	8.0	44.3	37.9	37.9	1.2:1:4.7
**BFO3/TO**	13.4	8.3	45.8	39.9	32.5	1.6:1:4.8
**BFO1/T**	12.9	10.2	50.3	45.6	26.6	1.3:1:4.5
**BFO2/T**	13.3	8.7	46.5	41.0	31.5	1.5:1:4.7
**BFO3/T**	13.7	7.5	44.1	37.5	34.7	1.8:1:5.0

Across all samples, the Bi, Fe, and O concentrations fall within the ranges typically reported for Bi–Fe–O oxide surfaces.^65–67^ The Bi/Fe ratios span ∼1.2–1.6 for TiO_2_-buffered samples and ∼1.3–1.8 for Ti-buffered films, indicating modest near-surface variations in cation proportions and a slightly Bi-rich surface relative to the ideal BiFeO_3_ stoichiometry (Bi/Fe/O = 1:1:3). Such ratios are consistent with previous XPS studies of polycrystalline BFO thin films.^4,67^ The O_r._ contents range from ∼38–46%at. for TiO_2_-buffered samples and ∼37–45%at. for Ti-buffered films, yielding O_r._/Fe ratios of ∼4.7–4.8 and ∼4.5–5.0, respectively. These elevated surface oxygen levels reflect the combined contributions of lattice oxygen and near-surface hydroxylated (−OH) species formed upon exposure to ambient.^54,67,68^

Comparison of the XPS compositional ratios with the XRD-derived Bi_2_O_3_ fractions reveals consistent trends across both buffer-layer series. For TiO_2_-buffered films, BFO2-TO (853 K, 5.2 × 10^–2^ mbar) exhibits the lowest Bi/Fe ratio and also the lowest Bi_2_O_3_ fraction from the XRD–RIR analysis. BFO1-TO (913 K) and BFO3-TO (793 K) show slightly higher Bi/Fe ratios, in agreement with their comparatively higher Bi_2_O_3_ fractions. A similar correspondence is observed for Ti-buffered samples: BFO1-T and BFO2-T show Bi/Fe ratios near ∼1.2–1.5, consistent with their lower relative Bi_2_O_3_ fractions, while BFO3-T exhibits the highest Bi/Fe ratio, matching its comparatively higher Bi_2_O_3_ fraction among the Ti-buffered series.

Although XPS cannot distinguish Bi originating from BiFeO_3_ versus Bi_2_O_3_ due to the close overlap in their binding energies,^68^ the overall Bi/Fe and O_r._ trends correlate well with the relative impurity fractions obtained from XRD. Samples with higher Bi_2_O_3_ fractions in XRD generally exhibit higher Bi/Fe ratios in XPS, whereas samples with lower Bi_2_O_3_ fractions show Bi/Fe ratios closer to unity. These compositional patterns complement the structural observations and morphological characteristics, demonstrating that both the buffer-layer chemistry and *P*_O_2__–*T*_S_ growth conditions contribute to systematic variations in the near-surface composition. Overall, the XPS survey results confirm that all films retain Bi–Fe–O chemistry consistent with BiFeO_3_, with subtle but systematic variations in elemental ratios that likely reflect the relative degree of parasitic Bi_2_O_3_ formation identified through diffraction and microstructural analysis.

To gain additional insight into the chemical state of each element and validate the oxidation-state assignments derived from survey spectra, high-resolution XPS fitting was performed for the Bi 4f, Fe 2p, O 1s, and C 1s regions. Figures 4 and 5 present high-resolution, normalized XPS spectra of the core levels for the BFOx-TO and BFOx-T films, respectively. All spectra were fitted using mixed Gaussian/Lorentzian line shapes [GL(30)] after Shirley background subtraction, consistent with established procedures for oxide systems.^37,69,70^ The fitting parameters, including peak binding energies, fwhm, and the relative area percentage (%Area) of the individual fitted components, are summarized in Table 3.

**Figure 4:**
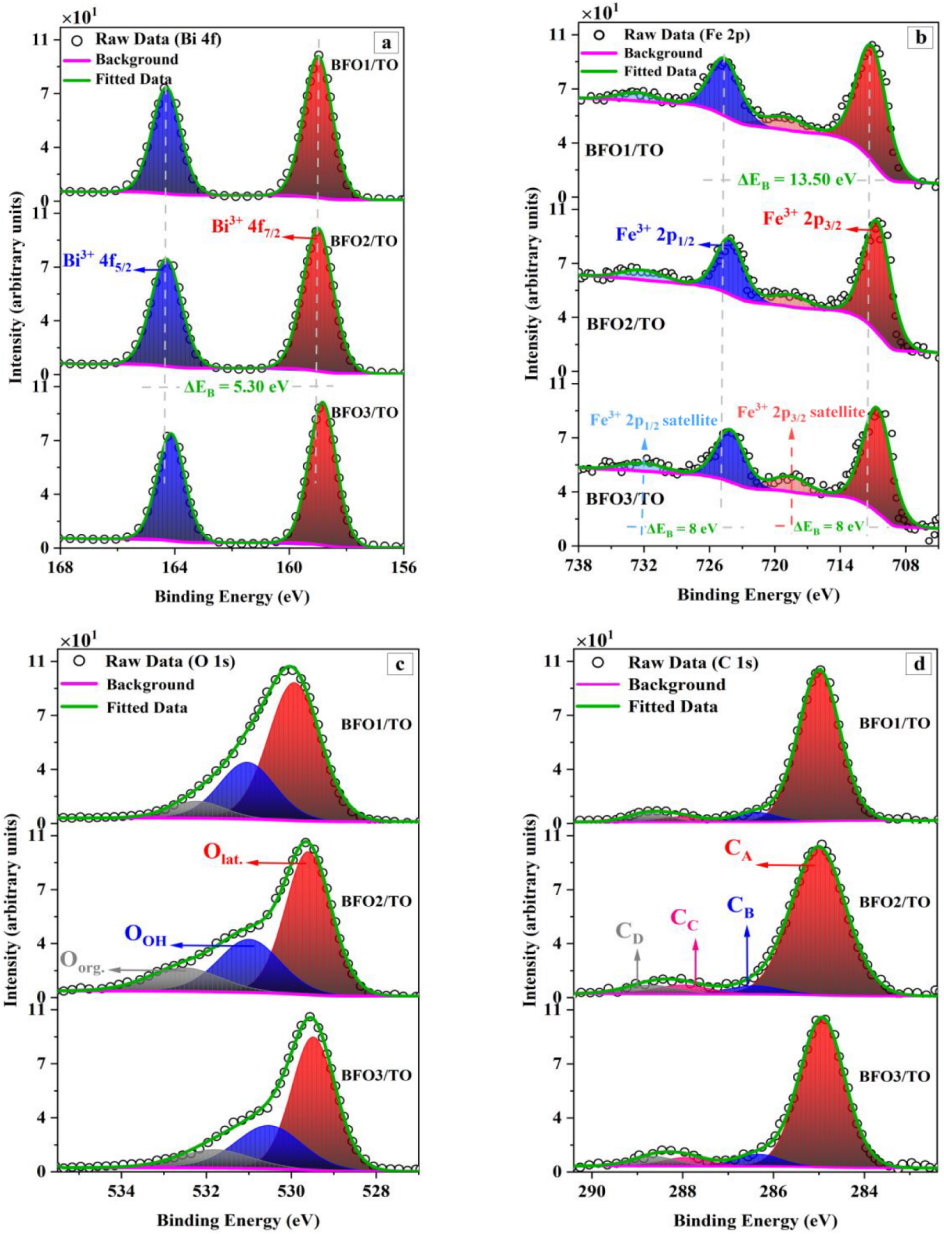
Normalized XPS high-resolution spectra of (a) Bi 4f, (b) Fe 2p, and (c) the O 1s core levels for BFO films deposited on the TiO_2_/Si substrate.

**Figure 5:**
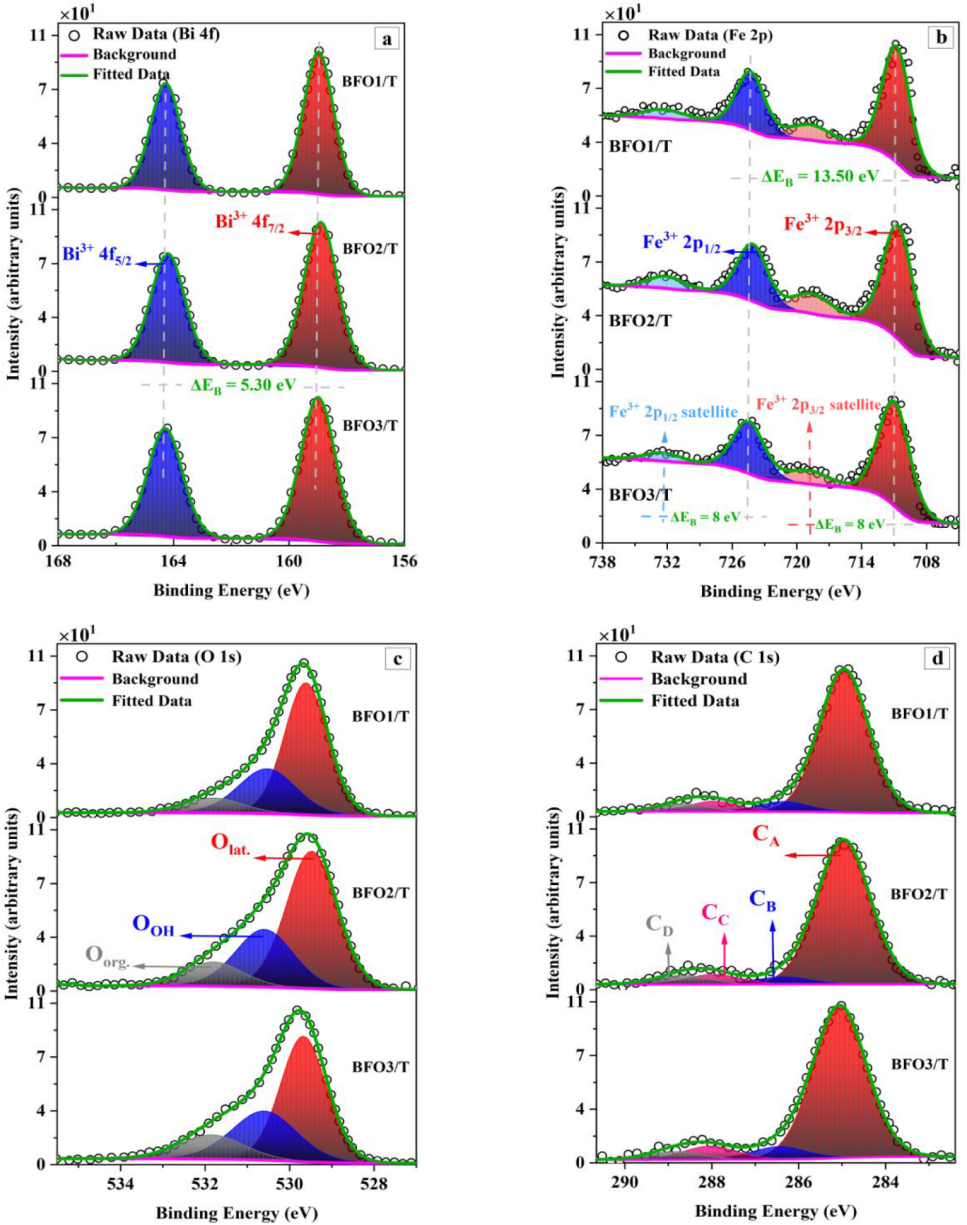
Normalized XPS high-resolution spectra of (a) Bi 4f, (b) Fe 2p, and (c) the O 1s core levels for BFO films deposited on the Ti/Si substrate.

**Table 3:** High-Resolution XPS Peak-Fit Parameters for the Bi 4f, Fe 2p, O 1s, and C 1s Core-Levels for BFOx-TO and BFOx-T Samples (Figures 4 and 5)­^a^

	**Peak**	**Bi 4f**		**Fe 2p**				**O 1s**			**C 1s**			
Sample		Bi^3+^ 4f_7/2_	Bi^3+^ 4f_5/2_	Fe^3+^ 2p_3/2_	Fe^3+^ 2p_1/2_	2p_3/2_ sat.	2p_1/2_ sat.	O_lat._	O_OH_	O_org._	C_A_	C_B_	C_C_	C_D_
**BFO1/TO**	Position (eV)	158.99	164.29	711.11	724.61	719.11	732.61	529.93	531.0	532.22	285.0	286.40	288.0	288.76
	fwhm (eV)	1.18	1.18	3.29	3.29	4.0	4.0	1.43	1.61	1.72	1.07	1.07	1.07	1.07
	%_Area_	57.3	42.7	56.8	28.4	9.1	5.7	63.1	28.6	8.3	86.2	5.6	2.9	5.3
**BFO2/TO**	Position (eV)	159.0	164.30	710.62	724.12	718.62	732.12	529.60	531.03	532.51	285.01	286.41	288.01	288.71
	fwhm (eV)	1.25	1.25	2.98	2.98	4.0	4.0	1.26	1.66	1.83	1.39	1.39	1.39	1.39
	%_Area_	57.2	42.8	55.9	28.0	8.3	7.8	56.9	28.5	14.6	84.9	4.8	5.2	5.1
**BFO3/TO**	Position (eV)	158.83	164.13	710.60	724.10	718.60	732.10	529.48	530.64	532.0	284.93	286.33	287.93	288.63
	fwhm (eV)	1.13	1.13	3.10	3.10	4.0	4.0	1.20	1.80	1.93	1.16	1.16	1.16	1.16
	%_Area_	57.4	42.6	54.8	27.4	10.8	7.0	58.0	28.9	13.1	83.0	6.7	5.0	5.3
**BFO1/T**	Position (eV)	158.97	164.27	710.77	724.27	718.77	732.27	529.61	530.63	532.01	284.95	286.35	287.95	288.65
	fwhm (eV)	1.23	1.23	3.08	3.08	4.10	4.10	1.19	1.64	1.75	1.33	1.33	1.33	1.33
	%_Area_	57.2	42.8	55.0	27.5	10.9	6.6	62.2	28.5	9.3	83.5	5.9	6.0	4.6
**BFO2/T**	Position (eV)	158.89	164.19	710.59	724.09	718.59	732.09	529.48	530.67	532.02	284.96	286.35	287.96	288.66
	fwhm (eV)	1.37	1.37	3.26	3.26	4.10	4.10	1.40	1.73	1.86	1.43	1.43	1.43	1.43
	%_Area_	57.1	42.9	53.5	26.7	12.5	7.3	59.6	28.6	11.8	84.7	4.3	5.8	5.2
**BFO3/T**	Position (eV)	158.99	164.29	710.95	724.45	718.95	732.45	529.67	530.70	532.05	285.05	286.45	288.05	288.87
	fwhm (eV)	1.41	1.41	3.40	3.40	4.10	4.10	1.28	1.81	1.97	1.45	1.45	1.45	1.45
	%_Area_	57.0	43.0	56.3	28.1	10.0	5.6	56.0	29.0	15.0	81.6	6.8	7.2	4.4

a All components were fitted using a GL(30) line shape with a Shirley background.

The high-resolution Bi 4f spectra for all films (Figures 4a and 5a) exhibit two sharp and symmetric peaks, well described by a single Bi^3+^ spin–orbit doublet. The Bi^3+^ 4f_7/2_ and Bi^3+^ 4f_5/2_ components, constrained to their expected 4:3 area ratio, appear at ∼158.94 ± 0.08 eV and ∼164.24 ± 0.08 eV for the BFOx-TO samples, and at ∼158.95 ± 0.05 eV and ∼164.25 ± 0.05 eV for the BFOx-T samples. The associated spin–orbit splitting (Δ*E*_B_) of ∼5.30 eV is consistent with literature values reported for Bi^3+^ in BiFeO_3_ and Bi_2_O_3_ reference systems.^4,6,11,55,61^

No lower- or higher-*E*_B_ contributions indicative of metallic Bi^0^ (≈156.8 eV), Bi^5+^ (≈160.5 eV), or suboxide states are detected within the spectral resolution.^68^ The absence of peak asymmetry or additional components confirms that all detectable Bi near the surface exists in the trivalent state, in agreement with the oxidized Bi environment inferred from XRD. Since the Bi 4f envelope contains only Bi^3+^ (i.e., %Area = 100%), the surface oxidation-state atomic fraction satisfies %at. (Bi^3+^) ≈ %at. (Bi)_survey_.^63,64^ Thus, the Bi atomic concentrations provided in Table 2 directly represent the Bi^3+^ surface composition for all *P*_O_2__–*T*_S_ deposition conditions and buffer-layer configurations.

The high-resolution Fe 2p spectra for all films (Figures 4b and 5b) are well described by a single Fe^3+^ spin–orbit doublet with its characteristic shakeup satellite. The Fe^3+^ 2p_3/2_ and Fe^3+^ 2p_1/2_ components, constrained to their expected 2:1 area ratio, appear at ∼710.78 ± 0.25 eV; ∼724.28 ± 0.25 eV for BFOx-TO films; and ∼710.77 ± 0.18 eV; ∼724.27 ± 0.18 eV for BFOx-T films. The observed Δ*E*_B_ of ∼13.50 eV and the high-*E*_B_ shakeup satellite positioned ∼8.0 eV above each main peak agree well with literature values for high-spin Fe^3+^ in BiFeO_3_ and related ferric oxides.^4,6,11,37,55^

No lower-*E*_B_ contributions attributable to Fe^0^ (≈705 eV) or Fe^2+^ (≤710 eV, with satellites ∼6 eV higher) are detected within the instrumental resolution.^68^ The absence of Fe^0^/Fe^2+^-associated asymmetry or additional components confirms that all detectable Fe at the near-surface exists in the trivalent oxidation state, consistent with the oxidized Fe environment of the perovskite structure indicated by XRD. Since only Fe^3+^ is present (i.e., %Area = 100%), the surface oxidation-state atomic fraction satisfies %at. (Fe^3+^) ≈ %at. (Fe)_survey_.^63,64^ Accordingly, the Fe atomic percentages in Table 2 directly represent the Fe^3+^ surface composition under *P*_O_2__–*T*_S_ deposition conditions and buffer-layer configurations.

It is well established that the Fe 2p spectrum in the oxide system displays intrinsic multiplet splitting, charge-transfer satellites, and complex final-state interactions. These multiplet structures do not correspond to individual chemical components but arise from 2p–3d exchange coupling of Fe cations.^71^ As highlighted in previous quantitative studies of Fe-oxide XPS, forcing a fit to multiple multiplet-resolved peaks can introduce nonunique, nonphysical fit parameters and does not improve chemical interpretation.^72,73^ In the present PLD-grown films, instrumental broadening, surface disorder, and limited signal-to-noise merge the multiplet features into a single broad envelope, making explicit multiplet-resolved fitting impractical.^37^ Therefore, following recommended oxide-XPS methodology, the Fe 2p region is fitted using a single Fe^3+^ spin–orbit doublet plus high-binding-energy shakeup satellites. This approach preserves the chemically relevant information (peak position, satellite (sat.) separation, and integrated Fe signal) while ensuring robust and reproducible fits across the full sample series. The corresponding fitting parameters are reported in Table 3.

The high-resolution O 1s spectra for all BFO films (Figures 4c and 5c) exhibit a multicomponent line shape characteristic of complex oxide surfaces. The dominant lattice-oxygen component (O_lat._) appears at ∼529.5–529.9 eV and is assigned to O^2–^ ions bonded to Bi and Fe within the BiFeO_3_ perovskite framework. This peak may also include contributions from the Bi_2_O_3_ phase identified by XRD, which overlaps in this binding-energy range. The position and shape of this component are consistent with reference spectra for BiFeO_3_ and Bi oxide systems reported in the literature.^4,68,74^

A second component located at ∼530.6–531.0 eV is attributed to surface hydroxylated oxygen (O_OH_). For oxide films measured ex situ, exposure to the ambient atmosphere leads to rapid surface hydroxylation; consequently, oxygen associated with −OH groups dominates the O 1s signal in this binding-energy range, as undercoordinated or dangling oxygen sites are readily converted to hydroxylated species. The reduced local screening and altered bonding environment associated with −OH species result in a positive chemical shift relative to fully coordinated lattice oxygen. Similar O 1s features assigned to surface −OH groups have been widely reported for BiFeO_3_ and oxide thin films.^4,64,74^

A higher-B_E_ contribution (O_org._) is detected at ∼532.0–532.5 eV and is attributed to carbon–oxygen species such as alcohol/ether, carbonyl, or carboxyl/ester carbonate functional groups originating from ambient exposure. These groups do not correspond to intrinsic BFO structure but instead result from surface-adsorbed contaminants commonly observed in ex-situ XPS analysis of oxide films.^4,63,68^

To isolate the film-related oxygen contributions, the fraction of O_org._ was quantified following the same AdC–oxygen correction method used for the survey spectra.^63^ Specifically, O associated with AdC species, %at. O_AdC_, was estimated using the total survey carbon, %at. C, and the relative area of oxygen-containing components in the C 1s spectrum. This calculated value was normalized to the total survey oxygen, %at. O, and converted into the expected O 1s contribution due to O_org_. The resulting value was used to constrain the high-resolution O 1s spectrum fitting, and the resulting relative area percentages for O_lat._, O_OH_, and O_org._ are reported as %Area values in Table 3. This correction step improves the accuracy of oxygen chemical-state interpretation by separating contributions from atmospheric carbon–oxygen species, allowing O_lat._ and O_OH_, to more reliably represent the intrinsic oxygen coordination and near-surface structure of the BFO films.

The high-resolution C 1s spectra for all BFO films (Figures 4d and 5d) were fitted into four chemically distinct components consistent with established models for AdC on oxide surfaces.^63^ The dominant component (C_A_) at ∼284.8–285.0 eV corresponds to C–C/C–H bonding environments and represents hydrocarbon contamination used for charge referencing. As this component does not contain oxygen, it does not contribute to the oxygen-quantification analysis.

Component (C_B_) at ∼286.3–286.5 eV is assigned to C–O species such as alcohols and ethers, while component (C_C_) at ∼287.8–288.0 eV corresponds to carbonyl (C=O) groups. The highest-binding-energy component (C_D_) at ∼288.6–288.9 eV arises from O—C=O environments associated with carboxyl or ester carbonate species. These oxygen-containing components (C_B_–C_D_) collectively reflect the degree of oxygenation within the AdC overlayer. Accordingly, their relative %Area was used to estimate the contribution of organic oxygen from AdC in both the survey and high-resolution O 1s spectra, enabling more reliable separation of intrinsic oxide-related oxygen from surface–adsorbate contributions.^63^

The high-resolution core-level analysis (Table 3) provides detailed insight into the near-surface oxidation states and oxygen coordination environments of the BFO films, enabling a direct comparison of buffer-dependent and *P*_O_2__–*T*_S_-dependent chemical trends. Across all deposition conditions, Bi and Fe are detected exclusively as Bi^3+^ and Fe^3+^, confirmed by the well-defined Bi 4f and Fe 2p spin–orbit doublets and their characteristic shakeup satellites. This uniformity indicates stable cation oxidation states throughout the series.

The O 1s region exhibits the most notable variations with deposition conditions. The O_lat._ component accounts for ∼56–63%Area across all films, indicating a predominantly oxidized Bi–Fe–O framework. A second component, O_OH_, contributes ∼28%Area for the films grown at *T*_S_ = 853 K and increases when the *T*_S_ is either raised or lowered. This behavior parallels the XRD-derived peak broadening and the evolution of Bi_2_O_3_ content, indicating that deviations from the intermediate-*T*_S_ growth condition promote increased surface hydroxylation and locally perturbed oxygen coordination.^63,64,68^ A third, higher-*E*_B_ contribution at ∼532–532.5 eV, associated with carbon–oxygen surface adsorbates, O_org._, remains ≤ 15%Area for all samples but shows modest increases away from the optimized growth regime. This trend is consistent with a larger effective surface area and enhanced adsorption of atmospheric species under less favorable growth conditions.^68^

Comparison between buffer layers shows that TiO_2_-buffered films often exhibit a slightly higher O_lat._ fraction and a marginally lower contribution from O_OH_ relative to Ti-buffered films under comparable *P*_O_2__–*T*_S_ conditions. These distinctions suggest a somewhat more compact and less hydroxylated near-surface structure on TiO_2_/Si, whereas Ti/Si surfaces display a modestly greater sensitivity to surface termination and atmospheric moisture uptake.^63,64,68^

To support these observations, the BFO surface compositions were additionally evaluated by deriving Bi/Fe/O ratios from the normalized surface atomic concentrations of the Bi^3+^, Fe^3+^, and lattice O^2–^ components, following procedures commonly adopted in prior XPS studies of oxide films.^63,64^ In addition, the contribution of nonlattice surface oxygen, dominated by hydroxylated environments, was evaluated using a semiquantitative proxy defined as the normalized area fraction of the O_OH_ component relative to the total O 1s envelope area (hereafter denoted as RIR).^4,64,75^ The resulting values are summarized in Table 4, enabling a comparative analysis of the buffer layer and *P*_O_2__–*T*_S_-dependent variations in near-surface stoichiometry and coordination.

Across both buffer-layer configurations (Table 4), the Bi^3+^/Fe^3+^ ratios deviate moderately from the nominal 1:1 stoichiometry, consistent with the presence of Bi_2_O_3_ detected by XRD. The normalized O^2–^ concentrations^64,75^ (i.e., %Area (O_lat._) weighted by the %at.(O)_survey_) fall within ∼24.7–32.0%at., giving O^2–^/Fe^3+^ ratios of ∼3.1–3.3 for most samples.^4,75,76^ These slightly elevated values relative to the ideal (3) reflect the contribution of oxidized oxygen in Bi_2_O_3_, whose O^2–^ B_E_ shift lies close to that of lattice oxygen in BiFeO_3_.^61,64^ Among the series, films grown under *T*_S_ ≈ 853 K and *P*_O_2__ = 5.2 × 10^–2^ mbar (BFO2-TO) and *T*_S_ ≈ 913 K and *P*_O_2__ = 1.3 × 10^–1^ mbar (BFO1-T) exhibit Bi^3+^/Fe^3+^/O^2–^ ratios closest to nominal BiFeO_3_ (∼1.2–1.3:1:3.1), indicating the most favorable cation coordination.

**Table 4:** Normalized Surface Compositions of Bi^3+^, Fe^3+^, and the O^2–^ Components for the BFOx-TO and BFOx-T Films^a^

	Normalized surface concentration (%at.)				
Sample	Bi^3+^	Fe^3+^	O^2^–^^	Bi^3+^/Fe^3+^/O^2^–^^	RIR
**BFO1/TO**	12.4	9.6	32.0	1.3:1:3.3	0.286
**BFO2/TO**	9.8	8.0	25.2	1.2:1:3.1	0.285
**BFO3/TO**	13.4	8.3	26.6	1.6:1:3.2	0.289
**BFO1/T**	12.9	10.2	31.3	1.3:1:3.1	0.285
**BFO2/T**	13.3	8.7	27.7	1.5:1:3.2	0.286
**BFO3/T**	13.7	7.5	24.7	1.8:1:3.3	0.290

a The Bi^3+^ and Fe^3+^ values correspond to the survey-derived cation concentrations, reflecting the exclusive presence of trivalent oxidation states in the Bi 4f and Fe 2p spectra. The O^2–^ values represent the normalized lattice-oxygen concentrations, calculated by weighting the %Area (O_lat._) with the %at.(O)_survey_. The RIR = O_OH_/(O_lat._ + O_OH_ + O_org._) quantifies the fraction of oxygen present in a hydroxylated near-surface environment.

More pronounced variations emerge in the XPS-derived RIR parameter. The lowest RIR values (∼0.284–0.285) are observed for BFO2-TO and BFO1-T, indicating reduced contributions from surface −OH species and a more ordered near-surface oxygen environment under these growth conditions.^37,64,74^ In contrast, samples grown outside this stability window (e.g., BFO3-TO/T) exhibit modestly higher RIR values (∼0.289–0.290), consistent with increased surface hydroxylation and enhanced exposure of reactive surface sites. These trends correlate with the higher Bi_2_O_3_ fractions identified by XRD–RIR analysis and reflect changes in surface chemistry.^55,64,68^

Taken together, the high-resolution XPS results are fully consistent with the structural and morphological observations. Films deposited at intermediate-to-high *T*_S_ combined with moderate-to-low *P*_O_2__ exhibit the most stoichiometric and chemically stable near-surface oxygen environments irrespective of buffer-layer selection. Within this deposition window, both TiO_2_- and Ti-buffered films show comparably low contributions from hydroxylated oxygen species and reduced Bi surface enrichment, in agreement with the improved crystallinity and minimized Bi_2_O_3_ content identified by XRD. Thus, the combined evidence identifies this *P*_O_2__–*T*_S_ regime as the optimal growth window for stabilizing BiFeO_3_ on Si, with the lowest degree of impurity-phase formation and the least disruption of near-surface oxygen coordination.

Previous studies on BiFeO_3_ thin films have established that *P*_O_2__ and *T*_S_ strongly influence the phase stability and oxygen coordination. Elevated *T*_S_ is generally associated with increased Bi volatility, whereas low *P*_O_2__ and/or reduced *T*_S_ can promote nonideal oxygen coordination and local chemical disorder at the film surface; conversely, higher *P*_O_2__ tends to favor oxygen-rich surface terminations and enhanced hydroxylation.^7,13^ Buffer layers further modulate these effects by altering cation volatility, oxygen-exchange kinetics, and strain accommodation during growth. Oxide buffers such as SrRuO_3_ and SrTiO_3_ have been reported to stabilize Bi^3+^/Fe^3+^ oxidation states and suppress Bi segregation, while metallic buffers can modify local redox conditions and thereby influence volatility-driven disorder and surface chemistry.^5,39,77^ Within this broader context, this study provides a systematic comparison of Ti- and TiO_2_-buffered BFO films grown under identical *P*_O_2__–*T*_S_ conditions. The combined XRD, SEM, and XPS analyses demonstrate that each buffer layer imposes characteristic constraints on phase evolution and near-surface chemical environments, enabling a detailed mapping of impurity formation and oxygen-coordination stability across the deposition window.

Rather than focusing solely on achieving phase-pure BFO, the persistence and evolution of Bi_2_O_3_ in this work are treated as diagnostic indicators that delineate the “impurity stability window.” Tracking these trends reveals a consistent regime: moderate-to-low *P*_O_2__ combined with intermediate-to-high *T*_S_, where impurity formation, nonlattice surface oxygen contributions, and cation-stoichiometry deviations are simultaneously minimized. Although parasitic phases do not vanish entirely within the studied conditions, their systematic variation provides predictive insight into the growth space most favorable for stabilizing BiFeO_3_ on Si.

This defect-informed perspective underscores how monitoring impurity-assisted pathways and surface-chemical signatures can be as informative as directly observing phase-pure formation, offering a practical strategy for defining stability limits in complex oxide thin films. Under optimized *P*_O_2__–*T*_S_ conditions, both TiO_2_ or Ti buffering promote improved structural stability and reduced surface-chemical disorder, supporting reliable integration of BiFeO_3_-based heterostructures for multifunctional electronic and spintronic applications.

## 4 Conclusions

This study demonstrates that buffer-layer chemistry plays a meaningful role in shaping the structural and surface-chemical characteristics of BiFeO_3_ films grown on Si. Across the explored *P*_O_2__–*T*_S_ range, TiO_2_-buffered films exhibit comparatively narrower diffraction peaks and smaller Bi/Fe variations, whereas Ti-buffered films show broader reflections, slightly lower Bi_2_O_3_ fractions under certain growth conditions, and more uniformly packed grain morphology. These results indicate that both TiO_2_- and Ti-based oxide buffers provide compositionally stable interfaces for BFO formation within the investigated deposition range.

Combined XRD, SEM, and XPS analyses identify a reproducible growth window, *T*_S_ ≈ 853 K at *P*_O_2__ = 5.2 × 10^–2^ mbar, where both buffer systems exhibit their lowest Bi_2_O_3_ fractions and the closest approach to nominal BiFeO_3_ surface stoichiometry. Outside this regime, deviations in Bi/Fe ratios, peak broadening, and higher Bi_2_O_3_ fractions become more pronounced at both lower and higher substrate temperatures, underscoring the joint influence of oxygen pressure and temperature on accessible phase stability.

In this work, the persistence of Bi_2_O_3_ across multiple deposition conditions is interpreted not as a limitation but as a diagnostic indicator for mapping an “impurity stability window.” Tracking its evolution allows identification of growth conditions that minimize parasitic phases while maintaining a consistent cation stoichiometry. The integrated structural and chemical evidence supports that intermediate-to-high *T*_S_ combined with moderate-to-low *P*_O_2__ provide the broadest conditions for achieving near-stoichiometric BiFeO_3_ surface chemistry. This framework offers practical guidance for selecting buffer architectures and growth parameters for BFO/Si integration.

Electrical resistivity or transport measurements were not performed in the present study, and no correlations are made between impurity content, Bi/Fe ratios, or oxygen-coordination states and electronic behavior. Future work will incorporate patterned test structures and four-point or J–V measurements to directly establish how buffer selection, surface composition, and impurity-phase evolution affect transport properties.
